# Sentinel lymph node biopsy for high-risk cutaneous squamous cell carcinoma: clinical experience and review of literature

**DOI:** 10.1186/1477-7819-9-80

**Published:** 2011-07-19

**Authors:** Steve Kwon, Zhao Ming Dong, Peter C Wu

**Affiliations:** 1 Department of Surgery, University of Washington, Seattle, WA, USA; 2 Department of Pathology, VA Puget Sound Health Care System, Seattle, WA, USA; 3 Department of Surgery, VA Puget Sound Health Care System, Seattle, WA, USA

**Keywords:** sentinel lymph node, squamous cell carcinoma, cutaneous, staging

## Abstract

High-risk cutaneous squamous cell carcinoma (SCC) is associated with an increased risk of metastases. The role of sentinel lymph node (SLN) biopsy in these patients remains unclear. To address this uncertainty, we collected clinical data on six patients with clinical N0 high-risk SCC that underwent SLN biopsy between 1999 and 2006 and performed a literature review of SLN procedures for SCC to study the utility of SLN biopsy. There were no positive SLN identified among six cases and there was one local and one distant recurrence on follow-up. Literature review identified 130 reported cases of SLN biopsy for SCC. The SLN positivity rate was 14.1%, 10.1%, and 18.6%; false negative rate was 15.4%, 0%, and 22.2%; and the negative predictive value was 97.8%, 100%, and 95.2% for all sites, head/neck, and truncal/extremity sites, respectively. SLN biopsy remains an investigational staging tool in clinically node-negative high-risk SCC patients. The higher false negative rate and lower negative predictive value among SCC of the trunk/extremity compared to SCC of the head/neck sites suggests a more cautious approach when treating patients with the former. Given the paucity of long-term follow up, an emphasis is placed upon the need for close surveillance regardless of SLN status.

## Introduction

Cutaneous squamous cell carcinoma (SCC) is overall the second most common skin cancer with approximately 200,000 new cases diagnosed each year in the U.S. and accounts for nearly 25% of annual skin cancer deaths [1-4]. Fortunately, the majority of cases is associated with a favorable prognosis and is often curable by surgical or local destructive therapy. However, a small subset of SCC tumors can be characterized by aggressive biologic behavior with an increased risk of locoregional recurrence and distant metastases. Numerous studies have identified high-risk factors in SCC patients [5-7] associated with a worse prognosis including large size, rapid growth rate, irregular borders, moderate/poor differentiation, perineural invasion, recurrent lesions, sites of prior radiotherapy or chronic inflammation, immunocompromised states, and genetic disorders including albinism and xeroderma pigmentosum. In terms of size and location, SCC tumors are considered high-risk when measuring greater than 2 cm on the trunk and extremities; > 1 cm on the cheeks, forehead, scalp and neck; and > 0.6 cm on the "mask areas" of the face, genitals, hands and feet. More recent studies have suggested that tumor thickness (Clark's level IV), desmoplastic growth, and development of nodal metastases are the strongest predictors for survival resembling cutaneous melanoma [8,9]. Patients with cutaneous SCC associated with high-risk tumor features reportedly have a higher rates of local recurrence ranging between 10-47.2%, and rates of regional and distant metastases between 11-47.3% [5,10].

Prognosis is generally poor in patients who develop nodal metastases with an expected 5-year survival of 26-34% and a 10-year survival rate of only 16%, underscoring the importance of early detection and treatment [5,10]. Recognizing that SCC typically spreads first to regional lymph nodes prior to the development of distant metastases [10-12], there may be a beneficial role to identify subclinical nodal metastasis for prognostic staging and guide further therapy including therapeutic lymph node dissection and adjuvant radiation. Currently, there is no consensus agreement on the standard of care staging practice for patients with high-risk cutaneous SCC.

Sentinel lymph node (SLN) biopsy has been widely accepted as a minimally invasive and highly accurate technique for detecting occult nodal metastases in breast cancer and cutaneous melanoma and has been validated as an independent prognostic factor for survival [13-17]. The utility of SLN biopsy for the staging of cutaneous SCC remains unproven and there is a lack of evidence-based practice guidelines. We contribute our institutional experience with SLN biopsy in patients diagnosed with high-risk cutaneous SCC and perform a review of current medical literature to define the predictive value and role of SLN biopsy in the management of occult nodal metastases from cutaneous SCC.

## Materials and methods

We reviewed our cumulative experience with SLN biopsy in patients diagnosed with high-risk cutaneous SCC undergoing surgical treatment between 1/1/1999 and 12/31/2006 at the VA Puget Sound Health Care System and the University of Washington Medical Center. Institutional review board approval was obtained from both institutions to conduct this retrospective study. Data were collected based upon retrospective review of the medical record and institutional tumor registry. A total of 6 patients were identified with clinically node-negative cutaneous squamous cell carcinoma associated with at least two high-risk features as shown in Table 1. The diagnosis of SCC was verified on histological examination and all patients had no clinical evidence of nodal metastases on physical examination or imaging studies.

**Table 1:** Patient characteristics, sentinel lymph node results, and followup status.

Patient	Age	Sex	Primary Site	High Risk Features*	SLN region	SLN #	SLN status	Excision Margins	Adjuvant Therapy	Follow up Time (mos)	Recurrence
1	51	M	forearm	a, c	axilla	1	neg	neg	no	1.3	no
2	76	M	chest wall	a, c	axilla	2	neg	neg	no	2.6	no
3	75	M	temporal	a, c, e, f	parotid	1	neg	4 mm	no	15.5	yes, local
4	89	F	temporal	a, e, g	parotid	3	neg	neg	no	11.8	no
5	67	M	upper arm	d, e	axilla	2	neg	neg	no	8.5	no
6	73	M	perineum	a, e, f	inguinal	2	neg	neg	no	12.8	yes, distant

* High risk features defined below:

a = size ≥20 mm (trunk/extremities), size ≥10 mm (head), size ≥6 mm (face, genitalia, hand/feet).

b = poorly defined borders

c = recurrent lesion

d = immunosuppression

e = moderate or poorly differentiated

f = rapidly growing

g = perineural involvement

All patients underwent preoperative lymphoscintigraphy using technetium-labeled sulfur colloid. Skin landmarks were marked to assist intraoperative SLN localization. Lymphazurin 1% isosulfan blue was injected intradermally surrounding the primary tumor site at the beginning of the procedure in 4 of 6 SCC patients. Two patients with cutaneous SCC lesions of the head and face did not undergo intraoperative blue dye injection. A small skin incision was made overlying the SLN location as determined by preoperative lymphoscintigraphy and intraoperative hand-held gamma probe guidance. All SLNs and any additional palpable nodes were harvested for pathologic examination. Surgical excision of the primary tumor was performed in 5 patients with a minimum 1 cm wide margin. One patient with a recurrent SCC of the temple was excised with a 0.4 cm narrow margin due to anatomic constraints. Submitted candidate sentinel lymph nodes were step-sectioned with the microtome at intervals of 150 micrometers (um) and examined under light microscopy with conventional H&E staining. Three patients underwent additional immunohistochemical staining using a pancytokeratin marker.

We conducted a literature review of sentinel lymph node procedures performed for the primary diagnosis of cutaneous SCC. The Medline, Ovid and Cochrane Library databases were searched using the following terms: sentinel lymph node, squamous cell carcinoma, cutaneous. All publications available in English were reviewed and data recorded including: number of cutaneous SCC cases, SLN results, adjuvant treatments, and follow up status. Using these cumulative results, we evaluated the utility of SLN biopsy to predict nodal disease/recurrence and excluded those studies without follow up information for this analysis. We calculated the probability of sentinel lymph node positivity, based upon the total number of patients undergoing successful SLN biopsy for all sites, head/neck, and truncal/extremity sites. The accuracy of SLN could not be assessed since completion lymph node dissection (LND) was not routinely performed following negative SLN biopsy. Previous studies in melanoma have also applied SLN failure rate, which is defined as the percentage of recurrences in the SLN-negative biopsied nodal basins, to estimate the overall rate of SLN biopsy failure to detect regional spread of the disease [14]. We also calculated the SLN failure rate for high-risk cutaneous SCC. The false negative rate, as defined in previous studies [18,19] as the rate of nodal recurrences to the number of false negative and true positive SLN cases, was also calculated along with the negative predictive value.

## Results

**Figure 1:**
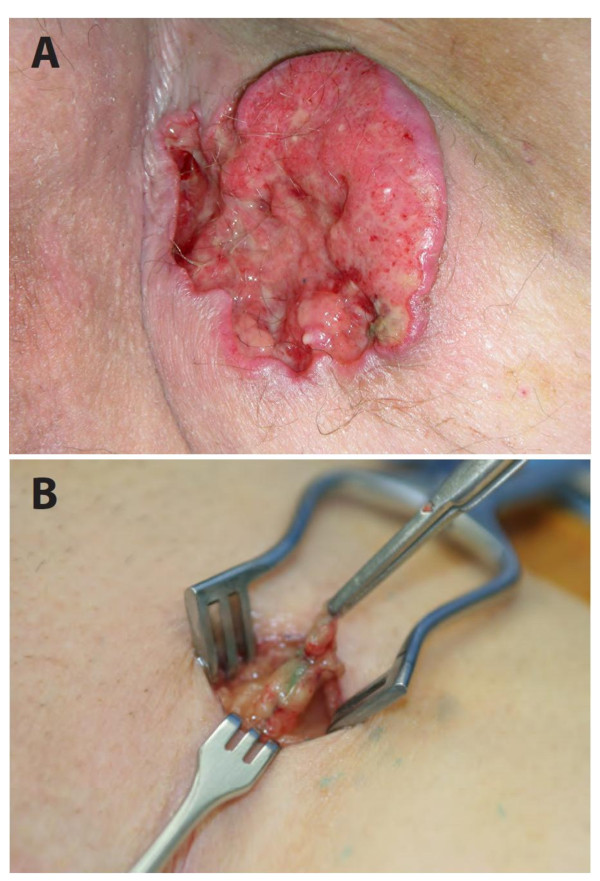
**(A) High-risk invasive perineal squamous cell carcinoma (B) Blue-stained inguinal sentinel lymph node**.

**Figure 2:**
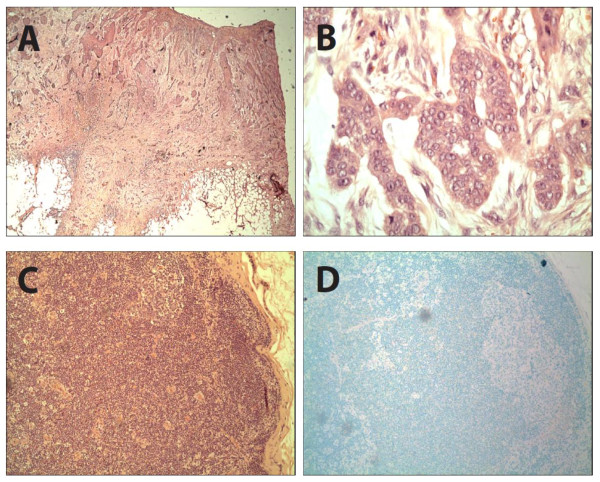
**Wide excision of perineal squamous cell carcinoma: H&E staining at 2X (A) and 40X (B)**. Sentinel lymph node biopsy: H&E at 10X (C) and immunostaining with pancytokeratin at 10X (D) showing no evidence of occult metastasis.

Six patients (5:1, M:F) with high-risk cutaneous SCC underwent SLN biopsy (mean age = 72 years, range 51-89 years). All patients had at least two previously described high-risk factors, two patients had 3 high-risk factors, and one patient had 4 high-risk factors. One patient developed a cutaneous SCC of the extremity during immunosuppression following successful heart transplantation. Mean tumor size in this case series was 3.2 cm (range: 1.3- 7 cm) and were located on the extremities (n = 2), head/face (n = 2), chest wall (n = 1) and perineum (n = 1, Figure 1). Three patients were referred for recurrent SCC tumors that had been previously treated within one year prior to the SLN procedure. Preoperative lymphoscintigraphy was performed in all 6 patients and identified 10 suspected SLNs. Intraoperative blue dye injection was used in 4 patients with extremity, truncal and perineal lesions. SLN exploration identified a combined total of 11 SLNs (median: 1.7 nodes per patient; range 1-3) as shown in Table 1. Upon pathologic examination with conventional H&E staining, there was no evidence of metastatic carcinoma in any of the submitted lymph nodes. Immunostaining was performed with pancytokeratin in three cases which showed no evidence of micrometastatic disease (Figure 2). There were no surgical complications following wide excision and SLN biopsy.

None of the patients received further adjuvant therapy and no completion LNDs were performed following negative SLN biopsy. Four patients are alive without evidence of disease progression after a median follow up of 10.1 months (range 1.3 - 15.5 months). One patient with a high-risk recurrent SCC of the right temple developed a second local recurrence 15.2 months following narrow-margin excision with negative SLN biopsy. A second patient with a high-risk large and deep perineal SCC developed metastatic lesions in the lung and vertebral bone 6.6 months after undergoing negative wide margin excision and negative SLN biopsy.

A review of the literature identified a total of 161 worldwide patients in 14 case series including this study [9,10,20-30], and 5 case reports [31-35] describing the use of SLN biopsy in patients with cutaneous SCC. Three case series [27-29] and one case report [31] were excluded since these patients were later combined into larger institutional case series resulting in a total of 130 evaluable cases (Table 2). All of the studies, except Hatta et al. [30] clearly designated cutaneous SCC cases with at least one high-risk feature. SLNs were successfully identified in 128 cases (98.5%). The probability of SLN positivity for all sites, head/neck, and truncal/extremity sites was found to be 14.1%, 10.1% and 18.6%, respectively. An evaluation of SLN outcomes from all available studies was performed (Table 3). Three studies [20,22,30] did not provide follow up status after SLN biopsy and only three studies [9,21,34] had a median follow up exceeding 2 years. A total of 100 SCC patients in 12 studies who underwent SLN biopsy had useful follow up information. Despite this limitation, an analysis of all documented recurrences showed an overall negative predictive value (NPV) of 97.8% for SLN status in high-risk patients. Among the head and neck cases (n = 51), the NPV for SLN biopsy was 100%, i.e. there were no regional nodal recurrences in any patient found to have a negative SLN. On the other hand, SLN biopsy for patients with high-risk lesions of the trunk and extremities (N = 49) had a noticeably lower NPV of 95.2%. Two patients in this high-risk group developed recurrent nodal disease despite undergoing a negative SLN biopsy. Also of note, there were two patients who relapsed with distant metastases despite a negative SLN biopsy (not included for NPV calculation).

**Table 2:** Summary of studies reporting SLN procedures for cutaneous squamous cell carcinoma.

Author, year	# SCC cases	Location	SLN results and histological methods	Adjuvant Treatment	Disease Recurrence
Stadelmann, 1997 [36]	1	Extremity	1/1 (100%), H&E	LND	LR (n = 1, +SLN)
Weisberg, 2000 [37]	1	Head	0/1 (0%), H&E and IHC	XRT	none
Altinyollar, 2002 [23]	20	Head	3/18 (17%), H&E	LND	N/A
Reschly, 2003 [10]	9	Head, Truncal/Extremity	4/9 (44%), H&E and IHC	LND (n = 3), XRT (n = 1)	LR (n = 1, +SLN), DR (n = 1, +SLN)
Michl, 2003 [24]	9	Head, Truncal/Extremity	2/9 (22%), H&E and IHC	LND + CTX/XRT (n = 2)	DR (n = 1, +SLN)
Eastman, 2004 [25]	6	Extremity	4/6 (67%). H&E and IHC	N/A	N/A
Ozcelik, 2004 [38]	1	Extremity	0/1 (0%), H&E	none	none
Wagner, 2004 [26]	12	Head, Truncal/Extremity	2/12 (17%), H&E	XRT (n = 2)	none
Hatta, 2005 [34]	4	Extremity	0/4 (0%), H&E and IHC	N/A	N/A
Perez-Naranjo, 2005 [39]	1	Extremity	0/1 (0%), N/A	none	none
Nouri, 2006 [27]	15	Head	1/15 (6.7%), H&E and IHC	LND (n = 4)	none
Mullen, 2006 [9]	14	Truncal/Extremity	0/14 (0%), H&E + IHC	none	LR (n = 2, -SLN) NR (n = 1, -SLN)
Sahn, 2007 [29]	9	Head, Truncal/Extremity	0/9 (0%), H&E and some IHC	XRT (n = 3)	NR (n = 1, -SLN) DR (n = 1, -SLN)
Renzi, 2007 [30]	22	Head, Truncal/Extremity	1/22 (5%), H&E and IHC	LND (n = 1)	DR (n = 1, +SLN)
Kwon, 2010	6	Head, Truncal/Extremity	0/6 (0%), H&E and some IHC.	none	LR (n = 1, -SLN) DR (n = 1, -SLN)

H&E = hematoxylin and eosin, IHC = immunohistochemistry

LND = lymph node dissection, XRT = radiation therapy, CTX = chemotherapy

LR = local recurrence, NR = nodal recurrence, DR = distant metastases, N/A = not available

**Table 3:** Cumulative results of sentinel lymph node (SLN) biopsy for high-risk cutaneous squamous cell carcinoma

	All sites	Head/Neck	Truncal/Extremity
# total cases	130	71	59
# total cases with identified SLN	128	69	59
# cases with SLN follow up	100	51	49
# cases with +SLN	18	7	11
# cases with +SLN and follow up	11	4	7
# local recurrences (LR)	5	1	4
# nodal recurrences (NR)	2	0	2
# distant recurrences (DR)	5	0	5
Rate of SLN positivity	14.1%	10.1%	18.6%
SLN failure rate*	2.2%	0%	4.8%
SLN negative predictive value	97.8%	100.0%	95.2%
SLN false negative rate†	15.4%	0%	22.2%

*defined as the percentage of recurrences in the SLN-negative biopsied nodal basins

†defined as the rate of nodal recurrences to the number of false negative and true positive SLN cases

The SLN failure rate was 2.2%. There were no false-negative SLN among the group of head/neck SCC tumors, while two patients with truncal/extremity SCC developed nodal recurrences despite negative SLN biopsy resulting in a SLN failure rate of 4.8%. The false negative rate was found to be 15.4% for all cases and 22.2% for the truncal/extremity group.

## Discussion

Though metastases from SCC of the skin are uncommon with a cumulative incidence between 2-6%, high-risk skin lesions are reported to have metastatic rates exceeding 30% [2]. It has been shown that regional nodal involvement increases both the risk of recurrence and mortality [9]. Metastases from cutaneous SCC tend to spread first to regional nodal basins and generally appear within the first 2 years of follow up [36]. Aggressive surgical treatment has been shown to benefit selected patients with locoregionally confined advanced SCC and long term survivors have been reported following radical salvage resection and therapeutic LND, though complication and mortality rates were reported in one study to be as high as 42% and 11%, respectively [6,9]. The role for elective LND in high-risk SCC remains undefined with most studies limited to head and neck primary sites. For these reasons, SLN biopsy is an unproven and yet theoretically appealing surgical technique to accurately stage high-risk SCCs with minimal morbidity, identify early occult nodal disease and select patients that might benefit from therapeutic LND or other adjuvant therapy..

The optimal management of clinical N0 patients with cutaneous SCC remains unclear. It appears that the overall SLN positivity rate (14.1%) for high-risk SCC is comparable to studies of high-risk melanoma which ranges from 13.9% - 29.4% [18]. SLN failure rate, false negative rate and NPV for SCC also resemble rates described in numerous melanoma studies. The standardized use of serial sectioning and immunostaining has significantly improved staging results of occult lymph node metastases in melanoma patients with one group reporting improved SLN positivity rates from 17.2 to 34% [37]. However, the benefit of routine immunostaining with cytokeratin markers for SCC patients has not been established. Given the distinct morphologic appearance of SCC characterized by very large and clustered cells [10], routine immunohistochemistry may not provide additional benefit. In fact, none of the studies reporting a positive SLN (Table 2) described a case where cytokeratin markers identified micrometastases not readily apparent on conventional H&E staining.

Regional node involvement of SCC is associated with an increased risk of recurrence and decreased survival. LND is recommended for patients with regional lymph node disease, though there are no significant studies that have shown whether this impacts overall survival in SCC patients. In a larger series of patients from the M.D. Andersen Cancer Center [9], 52% of patients who underwent LND for SCC regional nodal disease (n = 23) had disease recurrence and 75% of these patients later developed distant metastases. Unfortunately, there are no published prospective studies comparing LND with close observation in patients with clinical N0 high-risk SCC. Further studies on the utility of SLN biopsy as well as survival benefit from undergoing an elective LND after a positive SLN biopsy are needed.

We found, compared to head/neck sites, there were increased false negative rate and lower NPV for high-risk SCC of the trunk and extremities. This may have been secondary to differences in important prognostic factors for metastasis such as tumor thickness, immunosuppresion, desmoplasia, and increased horizontal size [38]. This was not evaluable given that many studies lacked these information. We cannot rule out the possibility that there may be inherent tumor biology differences between the two sites, and suggest a more cautious approach when treating patients with high-risk SCC of the trunk and extremities. In addition, considering the relatively short follow up in the majority of studies, the calculated NPV of SLN biopsy may in fact be overestimated. Considering the rarity of this tumor and lack of long-term follow up in the majority of studies, including our study, a clear emphasis is placed upon the need for close surveillance regardless of the SLN status. This study and review of literature highlights the potential limitations of SLN biopsy for SCC and the critical importance of careful long-term follow-up in these high-risk patients.

Though cytokeratin immunostaining may not directly impact the sensitivity or specificity of SLN status, recent studies have suggested that other pathologic markers can provide additional insight into tumor biology and cancer prognosis. A prospective study of non-well-differentiated SCC and matched controls confirmed that tumor thickness is the strongest prognostic risk factor in these SCCs [39]. This study also identified the potential value of Ki-67 expression to predict recurrence. Ki-67 is a cell-cycle protein that is upregulated during cellular proliferation and has been shown to correlate with the differentiation status of skin cancers. There is ongoing research to identify novel tumor biomarkers to define cancer prognosis and promote individualized therapies.

## Conclusions

We conclude that SLN biopsy remains an investigational staging tool in clinically node-negative high-risk cutaneous squamous cell carcinoma patients. It is obvious that larger, prospective studies with longer follow-up times are needed to establish the efficacy of SLN biopsy and define the optimal treatment of occult nodal metastasis for high-risk cutaneous SCC. It is unlikely that a large randomized controlled trial can be accomplished considering the relative low incidence of high-risk SCC and long accrual period that would be required. An alternative approach would be to contribute and analyze large prospective databases to define the role and limitations of SLN biopsy in this unique subset of SCC patients. Meanwhile, it is incumbent upon treating physicians and teams to closely follow these high-risk patients at greater risk for recurrence whether they undergo SLN biopsy or not.

## Abbreviations list

CTX: chemotherapy; DFS: disease-free survival; DR: distant recurrence; H&E: hematoxylin and eosin; IHC: immunohistochemistry; LND: lymph node dissection; LR: local recurrence; N/A: not available; NPV: negative predictive value; NR: nodal recurrence; SCC: squamous cell carcinoma; SLN: sentinel lymph node.

## Competing interests

The authors declare that they have no competing interests.

## Authors' contributions

SK did the data collection and data analysis, reviewed the literature, and wrote the manuscript. ZD provided the pathology figures and legends. PW wrote the manuscript and supervised the work. All authors read and approved the final manuscript.
